# Mean-level correspondence and moment-to-moment synchrony in adolescent and parent affect: Exploring associations with adolescent age and internalizing and externalizing symptoms

**DOI:** 10.1017/S0954579422000062

**Published:** 2022-04-07

**Authors:** Lauren M. Henry, Kelly H. Watson, David A. Cole, Sofia Torres, Allison Vreeland, Rachel E. Siciliano, Allegra S. Anderson, Meredith A. Gruhn, Abagail Ciriegio, Cassandra Broll, Jon Ebert, Tarah Kuhn, Bruce E. Compas

**Affiliations:** 1 Psychology and Human Development, Vanderbilt University, Nashville, TN, USA; 2 Vanderbilt University Medical Center, Nashville, TN, USA

**Keywords:** adolescence, affect, internalizing and externalizing problems, parents, synchrony

## Abstract

Interactions with parents are integral in shaping the development of children’s emotional processes. Important aspects of these interactions are overall (mean level) affective experience and affective synchrony (linkages between parent and child affect across time). Respectively, mean-level affect and affective synchrony reflect aspects of the content and structure of dyadic interactions. Most research on parent–child affect during dyadic interactions has focused on infancy and early childhood; adolescence, however, is a key period for both normative emotional development and the emergence of emotional disorders. We examined affect in early to mid-adolescents (*N* = 55, *M*_age_ = 12.27) and their parents using a video-mediated recall task of 10-min conflict-topic discussions. Using multilevel modeling, we found evidence of significant level-2 effects (mean affect) and level-1 effects (affective synchrony) for parents and their adolescents. Level-2 and level-1 associations were differentially moderated by adolescent age and adolescent internalizing and externalizing symptoms. More specifically, parent–adolescent synchrony was stronger when adolescents were older and had more internalizing problems. Further, more positive adolescent mean affect was associated with more positive parent affect (and vice versa), but only for dyads with low adolescent externalizing problems. Results underscore the importance of additional research examining parent–child affect in adolescence.

The recognition, understanding, and regulation of emotions is important for healthy development and impairments in these processes are associated with the risk of internalizing and externalizing problems (e.g., Compas et al., 2017; Eisenberg et al., 2010; Houben et al., 2015; Southam-Gerow & Kendall, 2002). Although emotions are experienced within the individual, they often take place in and are shaped by interpersonal relationships. Parent–child relationships are a primary context in which children learn about the nature of emotions and acquire the skills needed to regulate their emotions, and emotional exchanges are a central feature of parent–child relationships (Tan et al., 2020). To a great extent, learning about emotions and the development of emotion regulation skills takes place in the context of direct, face-to-face interactions between parents and their children. In dyadic interactions, parents express their own emotions, react to the emotions of their children, and sometimes discuss the nature of emotions with their children (Eisenberg et al., 1998). These processes are present in the earliest phases of development and continue in important ways throughout adolescence (Bariola et al., 2011).

Children and adolescents experience and express positive and negative affect at levels similar to their parents, both across development and during discrete interactions (Halberstadt & Eaton, 2002). For example, through observational coding of dyadic play interactions across 4 years, Sallquist et al. (2010) found rank-order stability in the expression of positive affect in mothers and their young children. Describing mean (i.e., average) levels of parent and child affect provides insight into one aspect of the affective *content* of dyadic interactions (Lunkenheimer et al., 2011; Southam-Gerow & Kendall, 2002; Suveg et al., 2007). But what about their *structure*? In a treatment study of aggressive youth, De Rubeis and Granic (2012) found changes in mean affect during parent–child dyadic interactions from pretreatment to posttreatment to be unrelated to youth posttreatment externalizing symptoms. However, parent–child dyads with greater affective synchrony during interactions showed lower pretreatment levels of externalizing symptoms and demonstrated greater treatment gains compared to dysregulated dyads. In the current study, mean-level affect informs the content of parent–child interactions and affective synchrony provides insight into an important aspect of their dynamic structure. Examining both affective content and structure provides a more holistic picture of complex dyadic interactions and the best opportunity for elucidating the ways in which short-term affective exchanges relate to long-term youth emotional and behavioral functioning.

Understanding how systems change (and remain stable) is central to developmental psychopathology (Granic & Hollenstein, 2003), and extant research underscores the importance of examining the ways in which dyadic partners’ emotions are linked across time (Butler, 2011; Kuppens et al., 2010). Myriad terms have been used to describe this process (e.g., emotional or affective contingency, attunement, coregulation, reciprocity, responsiveness; Bornstein, 2013), and various statistical methods have been used to quantify moment-to-moment interpersonal coordination (e.g., linear regression, time series analysis, multilevel modeling, conditional probabilities; Butler, 2011). In the present study, we use the term *affective synchrony* to reflect the covariation of parent and adolescent affect across time. The earliest evidence of affective synchrony is found in the interactions of parents and their infants and young children (Bornstein et al., 2021; Feldman, 2007). In these developmental periods, higher levels of parent–child affective synchrony are related to better child self-control, social competence, and school performance, and less child aggression and social withdrawal (Cole et al., 2003; Feldman et al., 1999; Harrist et al., 1994; Lunkenheimer et al., 2020). Despite the importance of extending research on parent–child affective synchrony across development, surprisingly little research on this topic has focused on adolescence (Lougheed, 2020).

The nature of parent–child mean affect and affective synchrony may change over time and be particularly important over the course of adolescence. Adolescence is a critical transitional period. During childhood, a characteristic, predictable, and stable pattern of behavior develops between parent and child based on repeated interactions (Granic et al., 2003). In the transition from childhood to adolescence, close relationships with parents are maintained (Collins & Laursen, 2004) but entrenched dyadic interactions are perturbed as the family system negotiates new issues (e.g., autonomy, less predictable behavior and emotion; Granic et al., 2003). Conflict discussions are key contexts in which the vertical relationships of childhood (i.e., parents hold knowledge and power and provide security and warmth) become more horizontal (i.e., interactions are equal, symmetrical, and reciprocal; Branje, 2018), and so conflictual interactions may increase in frequency and intensity in adolescence (Granic et al., 2003). Accordingly, we might expect that mean (i.e., average) levels of parent and youth affect within conflict discussions become increasingly negative with adolescent age. However, the nature of the moment-to-moment coordination of parent and adolescent affect during conflict discussions is unclear. It is plausible to expect that parent–adolescent affective synchrony decreases across adolescence, as adolescents become more independent and autonomous. During conflict discussions, lower levels of parent–adolescent affective synchrony could also reflect efforts by parents to prevent escalation by maintaining neutral affect while adolescents have more extreme affective displays (van Bommel et al., 2019). Conversely, parent– adolescent affective synchrony may increase across adolescence, as adolescents mature and become more responsive to their parents’ emotions in constructive ways (Branje, 2018). During conflict discussions, higher levels of parent–adolescent affective synchrony could reflect emotional acceptance or empathy by parents (e.g., Creavy et al., 2020; Jospe et al., 2020).

Several disorders increase in prevalence during adolescence, including depression and some forms of disruptive behavior disorders (Kessler et al., 2007). Deficits in individual ability to regulate emotions is a key mechanism in the development of psychopathology (Compas et al., 2017; McLaughlin & Lambert, 2017; Yap et al., 2007), and emotion regulation skills are shaped in part through repeated exchanges with parents (Tan et al., 2020). Accordingly, both mean-level (i.e., average) affect and affective synchrony during parent–adolescent interactions may be related to adolescent symptoms of internalizing and externalizing disorders (Granic, 2005; Morris et al., 2018). Broadly, parental expressions of negative affect and behaviors (e.g., hostility, criticism) during parent–child interactions are linked to youth psychopathology (Peris & Miklowitz, 2015). In childhood, fewer behavioral problems have been observed in youth whose dyadic interactions with their parents are characterized by more positive affect and less negative affect (Lunkenheimer et al., 2011) and greater affective synchrony (Harrist et al., 1994; Kerr et al., 2021; Lougheed et al., 2015). Still, the ways in which mean-level affect and affective synchrony relate to youth internalizing and externalizing symptoms are not entirely clear, especially during adolescence. For example, Schwartz et al. (2014) found that maternal aggressive responses to both adolescent aggression (synchronous) and adolescent positive affect (asynchronous) are related to the onset of adolescent depression. While parent–adolescent interactions characterized by high and escalating levels of negative emotions may be associated with higher levels of adolescent internalizing and externalizing problems (Granic & Patterson, 2006; Yap et al., 2010), so might patterns of parental nonresponsiveness to adolescents’ emotions (Yap et al., 2008). Associations between affective synchrony and internalizing and externalizing symptoms during adolescence have not been a major focus of previous research (Lougheed, 2020).

The measurement of affective synchrony ranges from macro- to micro-level methods, including parent and adolescent self-reports of emotions over moments, weeks, months, and years (e.g., Kim et al., 2001) and observations and ratings of emotion by trained coders during parent–adolescent interactions (e.g., van Bommel et al., 2019). In terms of time scale, measurement of parent and adolescent affect *on a moment-to-moment basis* is arguably the most powerful and sensitive method to capture affective synchrony. With regard to reporting, both self-report and observational coding have methodological benefits and drawbacks. Observational coding by independent raters captures expressed emotion rather than experienced emotion and a large body of research has shown that these constructs are distinct (Ekman, 1972, 1993; Greenaway & Kalokerinos, 2019); both are important but inform separate research questions. Whereas self-reports capture subjective emotional experience, asking individuals to freely recall their affect after events occur may contribute to measurement error, including biases in retrospective recall (Feldman-Barrett, 1997; Kahneman, 2000; Rosenberg & Ekman, 1994).

A potentially valuable alternative method is video-mediated recall (VMR), which involves obtaining reports from members of dyads about their emotions while they view recordings of their face-to-face interactions (Welsh & Dickson, 2005). VMR can be conceptualized as an amalgam of self-report and observational coding giving rise to a set of advantages achieved by neither method alone. VMR allows for the use of specific, controlled tasks that provide similar contexts for emotions within and across parent–adolescent dyads (inherent to observational coding), informs internal, covert emotional processes that are not accessible to external observers (inherent to self-reports), and minimizes susceptibility to errors in recall as compared to retrospective reports over longer periods of time. VMR has been used successfully in studies of affective synchrony in married couples (Rohrbaugh et al., 2009) and randomly paired undergraduate students (Butler et al., 2014). VMR has also been used to understand individual differences in emotion dynamics in adolescents (Yang et al., 2019). To our knowledge, however, this approach has only been used in one study with parent–adolescent dyads (see Main et al., 2021).

The current study used VMR on a moment-to-moment basis to capture the dynamics of affect in interactions between parents and their children during a laboratory conflict-topic discussion task. Our sample of youth ranged from 10 to 15 years old, and accordingly we tested our hypotheses in early to mid-adolescence. We addressed the following three aims and four hypotheses. *Aim 1.* We examined the association between parent and adolescent affect over the course of the conflict-topic discussion task. As predictors, we used moment-to-moment changes in each partner’s affect^1^ as well as each partner’s mean level of affect. In the context of multilevel modeling, these associations represent the relation of variables within person but across time (level 1) and the relation of variables after pooling across time (level 2), respectively. In hypothesis one, we expected mean parent and adolescent affect to be positively related to partner affect. In hypothesis two, we expected positive associations between partners’ concurrent, moment-to-moment affect; in other words, we expected to observe affective synchrony. *Aim 2.* We explored whether affective processes in parent–child interactions differ as a function of adolescent age. For hypothesis three, we expected that parent and adolescent mean affect would be inversely related to adolescent age. Considering limited research on affective synchrony in adolescence, analyses examining the association of age and moment-to-moment affect were exploratory. *Aim 3.* We were interested in the ways in which associations between parent and adolescent affect differed based on levels of adolescent internalizing and externalizing symptoms. For hypothesis four, we expected that mean affect would be more negative for both parents and adolescents when levels of adolescent internalizing and externalizing symptoms were higher. Again, due to scant research on affective synchrony in adolescence, analyses examining the interaction between adolescent symptoms and moment-to-moment affect were exploratory.

## Method

### Participants and procedure

Parents and adolescents in a southern metropolitan area were recruited to participate in a study about stress and emotions in families from October 2018 to February 2020 (recruitment was suspended in March 2020 due to the COVID-19 pandemic). In order to enroll a sample that varied regarding adolescents’ emotional and behavioral problems, recruitment occurred primarily through listserv postings and outpatient clinic referrals. Parents who expressed interest in the study were screened on the phone prior to enrollment. Parent–adolescent dyads were excluded from participation if the adolescent was outside the age range of 10–15 years old, the parent was not the legal guardian of the adolescent, or the dyad did not live together at least 50% of the time for the past 6 months. Dyads were also excluded if mothers reported a diagnosis of schizophrenia in themselves or their adolescent or a diagnosis of autism spectrum disorder in the adolescent, as these disorders may impede an individual’s ability to complete some of the study tasks. In families with multiple adolescents meeting inclusion criteria, the older adolescent was invited to participate in the study.

Fifty-six dyads were enrolled and participated in the study. One dyad did not complete the conflict-topic discussion task due to an abbreviated lab visit, and their data were excluded listwise from the current analyses. Thus, 55 dyads contributed partial or full data to the current analyses. VMR data were missing from two parents and three adolescents due to research assistant error during the study visit (e.g., errors in installing the software or saving the data); no families refused to complete the VMR task. Continuous VMR affect ratings from the 55 dyads were divided into sixty 10-s intervals (see Data Analytic Strategy for details), resulting in 3,300 observations for analysis.

Adolescents were between 10 and 15 years old (*M* = 12.27; *SD* = 1.67), and 55% were female. Sixty-nine percent were White, 24% Black, 6% Asian, and 2% more than one race. Parents were primarily female (93%)^2^, married or living with a partner (69%), and ranged in education (37% completed a graduate degree, 9% some graduate education, 30% college graduate, 22% some college, 2% high school graduate). See Table 1 for additional information about sociodemographic characteristics of the sample. Parent–adolescent dyads completed a 4- to 5-hr laboratory visit, which included a series of questionnaires, interviews, and laboratory tasks. At the beginning of the visit, parents and adolescents independently completed an adapted version of the Issues Checklist (Robin & Foster, 1989). The Issues Checklist asks respondents to indicate which of 44 topics they discussed with their partner in the last 4 weeks (e.g., coming home on time, helping out around the house), and how they felt during those discussions (1 = *calm*, 5 = *angry*). Research assistants selected one topic that was rated highly by both the parent and adolescent. If the parent and adolescent did not rate the same topic highly, research assistants selected the topic that was rated highest by the adolescent. Dyads participated in a videotaped discussion task, during which they were instructed to discuss the selected topic for 10 min. The dyad was asked to (a) describe the issue, (b) explain how they feel about it, (c) discuss why it has become a source of conflict, and (d) attempt to resolve the issue. After the discussion, parents and adolescents viewed the videotape in separate rooms and provided moment-to-moment ratings of their own in-task affect (Girard, 2014).

Prior to the laboratory visit, adolescents completed the Youth Self Report (YSR; Achenbach & Rescorla, 2001) using REDCap survey software (Harris et al., 2009). Parents received $100 for their participation in the study, and adolescents received $50. All procedures were approved by the Vanderbilt University institutional review board.

### Measures

#### Adolescent and parent affect

Adolescents and their parents rated their in-task affect using a VMR procedure with Continuous Affect Rating and Media Annotation software (CARMA; Girard, 2014). CARMA is a media annotation program that displays audio and video files while collecting continuous ratings on a selected dimension. In the current study, adolescents and parents watched (with audio) their 10-min video recorded discussion task on a computer while continuously rating their own in-task affect using a joystick. Adolescents and parents completed this task in separate rooms. The affect slider displayed on the computer screen ranged from very negative (−100) to very positive (100). CARMA software samples the position of the joystick 10 times per second and computes second-by-second affect means. We averaged affect ratings across 10-s intervals within the 10-min task, creating 60 total ratings for each individual. Acceptable construct, concurrent, and discriminant validity has been established for VMR procedures (Lorber, 2007; Mauss et al., 2005).

#### Adolescent internalizing and externalizing symptoms

Adolescent internalizing and externalizing symptoms were measured using the YSR (Achenbach & Rescorla, 2001). The 112-item self-report checklist queries symptoms and behaviors over the past 6 months on a 3-point scale (0 = *not true*,1 = *somewhat or sometimes true*, and 2 = *very true or often true*). The reliability and validity of the YSR are well established (Achenbach & Rescorla, 2001). The present study used information from the Internalizing and Externalizing scales, which include 31 items on anxiety, depression, and somatic complaints and 32 items on rule-breaking and aggressive behaviors, respectively. In the current sample, internal consistency reliability for the Internalizing (α = 0.89) and Externalizing (α = 0.86) scales were good (Nunnally & Bernstein, 1994). Although the YSR is specified for 11- to 18-year-olds, guided by the definition of adolescence as the second decade of life (Lerner & Steinberg, 2004), 12 adolescents in the current sample were 10 years old. Internal consistency was adequate for this subgroup (Internalizing α = 0.92, Externalizing α = 0.67). Raw scores were used in analyses.

### Data analytic strategy

#### Planned analyses

We used SPSS Version 26 for all analyses. In preliminary analyses, we computed zero-order correlations to understand basic associations among the study variables. We used multilevel modeling to test all hypotheses. Full information maximum likelihood estimation was implemented in multilevel modeling analyses to account for missing data. First, intraclass correlation coefficients (ICC) were estimated in separate null models predicting parent affect and adolescent affect to establish need for multilevel modeling (Pornprasertmanit et al., 2014). See Supplementary Information for null model equations. Second, we conducted two sets of univariate analyses, one predicting adolescent affect as the dependent variable (*AA*_*td*_; henceforth referred to as the “Adolescent Affect Model”) and the other predicting parent affect as the dependent variable *PA*_*td*_; henceforth referred to as the “Parent Affect Model”). The Adolescent Affect Model is displayed below. See Supplementary Information for a parallel equation that reflects the Parent Affect Model.^3.^


$$\text{Level}\;1\;\text{equation:}\;AA_{td}=\beta_{0d}+\beta_{1}\left(PA_{td}-{\bar{PA}}_{.d}\right)+e_{td}$$

$$\text{Level}\;2\;\text{equation:}\;\beta_{0d}=\gamma_{00}+\gamma_{01}{\bar{PA}}_{.d}+u_{0d}$$

$$\beta_{1d}=\gamma_{10}+u_{1d}$$

$$\text{Mixed model:}\;AA_{td}=\gamma_{00}+\gamma_{01}{\bar{PA}}_{.d}+\gamma_{10}\left(PA_{td}-{\bar{PA}}_{.\text{d}}\right)\;\;+u_{0d}+u_{1d}\left(PA_{td}-{\bar{PA}}_{.d}\right)$$

$$e_{td}\sim N\left(0,\sigma_{e}^{2}\right)$$

$$\left[\begin{array}{l} u_{0d}\\ u_{1d} \end{array}\right]\sim N\left(\left[\begin{array}{l} 0\\ 0 \end{array}\right],\left[\begin{array}{ll} \tau_{00} & \\ \tau_{10} & \tau_{11} \end{array}\right]\right)$$

In the Adolescent Affect Model, parent affect ratings for all 10-s intervals were person-mean centered. In other words, each parent’s mean affect score across the 10-min interaction was subtracted from their affect ratings at each 10-s interval $\left(PA_{td}-{\bar{PA}}_{.d}\right)$. The parent person mean-centered affect score $\left(PA_{td}-{\bar{PA}}_{.d}\right)$ was included as a level-1 predictor, and the parent mean affect score $\left({\bar{PA}}_{.d}\right)$ was included as a level-2 predictor. Accordingly, $\gamma_{01}$ provides a test of hypothesis 1, the level-2 associations of mean affect. Further, $\gamma_{10}$ provides a test of hypothesis 2, the level-1 associations of affective synchrony. Third, we built upon our two sets of univariate analyses to address Aims 2 and 3. We included adolescent age, adolescent internalizing symptoms, and adolescent externalizing symptoms as level-2 predictors of level-1 slopes and intercepts. All slopes and intercepts were random effects. The Adolescent Affect Model is displayed below. See Supplementary Information for an equation that reflects the Parent Affect Model.


$$\text{Level}\;1\;\text{equation:}\;AA_{td}=\beta_{0d}+\beta_{1}\left(PA_{td}-{\bar{PA}}_{.d}\right)+e_{td}$$

$$\text{Level}\;2\;\text{equation:}\;\beta_{0d}=\gamma_{00}+\gamma_{01}{\bar{PA}}_{.d}+\gamma_{02}{\text{age}}_{d}+\gamma_{03}\;{\text{intern}}_{d}\;\;+\gamma_{04}\;{\text{extern}}_{d}+\gamma_{05}\;{\text{age}}_{d}{\bar{PA}}_{.d}\;\;+\gamma_{06}\;{\text{intern}}_{d}{\bar{PA}}_{.d}+\gamma_{07}\;{\text{extern}}_{d}{\bar{PA}}_{.d}\;\;+u_{0d}$$

$$\beta_{1d}=\gamma_{10}+\gamma_{11}{\text{age}}_{d}+\gamma_{12}{\text{intern}}_{d}+\gamma_{13}{\text{extern}}_{d}+u_{1d}$$

$$\text{Mixed model:}\;AA_{td}=\gamma_{00}+\gamma_{01}{\bar{PA}}_{.d}+\gamma_{02}{\text{age}}_{d}+\gamma_{03}{\text{intern}}_{d}\;\;+\gamma_{04}\;{\text{extern}}_{d}+\gamma_{05}\;{\text{age}}_{d}{\bar{PA}}_{.d}\;\;+\gamma_{06}\;{\text{intern}}_{d}{\bar{PA}}_{.d}+\gamma_{07}\;{\text{extern}}_{d}{\bar{PA}}_{.d}\;\;+\gamma_{10}(PA_{td}-{\bar{PA}}_{.d})\;\;+\gamma_{11}\;{\text{age}}_{d}(PA_{td}-{\bar{PA}}_{.d})\;\;+\gamma_{12}\;{\text{intern}}_{d}(PA_{td}-{\bar{PA}}_{.d})\;\;+\gamma_{13}\;{\text{extern}}_{d}(PA_{td}-{\bar{PA}}_{.d})+u_{0d}\;\;+u_{1d}\;(PA_{td}-{\bar{PA}}_{.d})$$

$$e_{td}\sim N\left(0,\sigma_{e}^{2}\right)$$

$$\left[\begin{array}{l} u_{0d}\\ u_{1d} \end{array}\right]\sim N\left(\left[\begin{array}{l} 0\\ 0 \end{array}\right],\left[\begin{array}{ll} \tau_{00} & \\ \tau_{10} & \tau_{11} \end{array}\right]\right)$$

## Results

### Descriptive statistics and bivariate correlation analyses

Table 2 displays means and standard deviations for study variables (adolescent affect, parent affect, adolescent internalizing symptoms, adolescent externalizing symptoms). For both adolescents and parents, affect ratings ranged from very negative (−100) to very positive (100), and the mean affect ratings were close to neutral (adolescent affect: *M* = 4.23, *SD* = 44.13; parent affect: *M* = 5.65, *SD* = 41.78). Distributions of adolescent and parent affect ratings were extremely similar. Approximately 10.1% of adolescent ratings and 10.1% of parent ratings were very negative (−100 to −50); 33.6% of adolescent ratings and 29.3% of parent ratings were moderately negative (−50 to −1); 9.6% of adolescent ratings and 9.2% of parent ratings were neutral (0); 31.3% of adolescent ratings and 36.3% of parent ratings were moderately positive (1 to 50); and 15.4% of adolescent ratings and 15.1% of parent ratings were very positive (51 to 100). Of the 43 participants for whom we were able to calculate symptom *T* scores (i.e., adolescents > 10 years old), adolescent symptoms were approximately one-half standard deviation above the normative mean for internalizing symptoms (*M*= 56.79, *SD* = 9.80) and externalizing symptoms (*M*= 53.05, *SD* = 9.93).^4^

Zero-order correlations are also presented in Table 2. As hypothesized, adolescent and parent affect were positively correlated with a medium effect. For adolescents, higher levels of internalizing and externalizing symptoms were associated with more negative in-task affect with small effects. Higher levels of adolescent internalizing and externalizing symptoms were associated with more negative in-task parent affect with small effects. As these correlations were in the expected directions, they provide evidence of construct validity of parent and adolescent affect as measured by VMR.

### Multilevel modeling

The ICC derived from the null univariate models predicting parent and adolescent affect indicated that 38% of the observed variation in parent affect and 48% of the observed variation in adolescent affect was due to differences among dyads (Table 3). These values suggest that multilevel modeling is an appropriate analytic method for these data.

Tests of hypotheses 1 and 2 (Aim 1) are presented in Table 4. In support of hypothesis 1 (level-2 analyses), multilevel models demonstrated that more positive mean parent affect ratings were associated with more positive adolescent affect ratings ($\gamma_{01}$ = .56, *p* = .001), and more positive mean adolescent affect ratings were associated with more positive parent affect ratings ($\gamma_{01}$ = .35, *p* = .001). In support of hypothesis 2 (level-1 analyses), multilevel models showed that more positive parent affect ratings were associated with more positive concurrent adolescent affect ratings ($\gamma_{10}$ = .24, *p* < .001), and more positive adolescent affect ratings were associated with more positive concurrent parent affect ratings ($\gamma_{10}$ = .28, *p* < .001). That is, results provide evidence of parent–child affective synchrony in adolescence.

**Figure 1:**
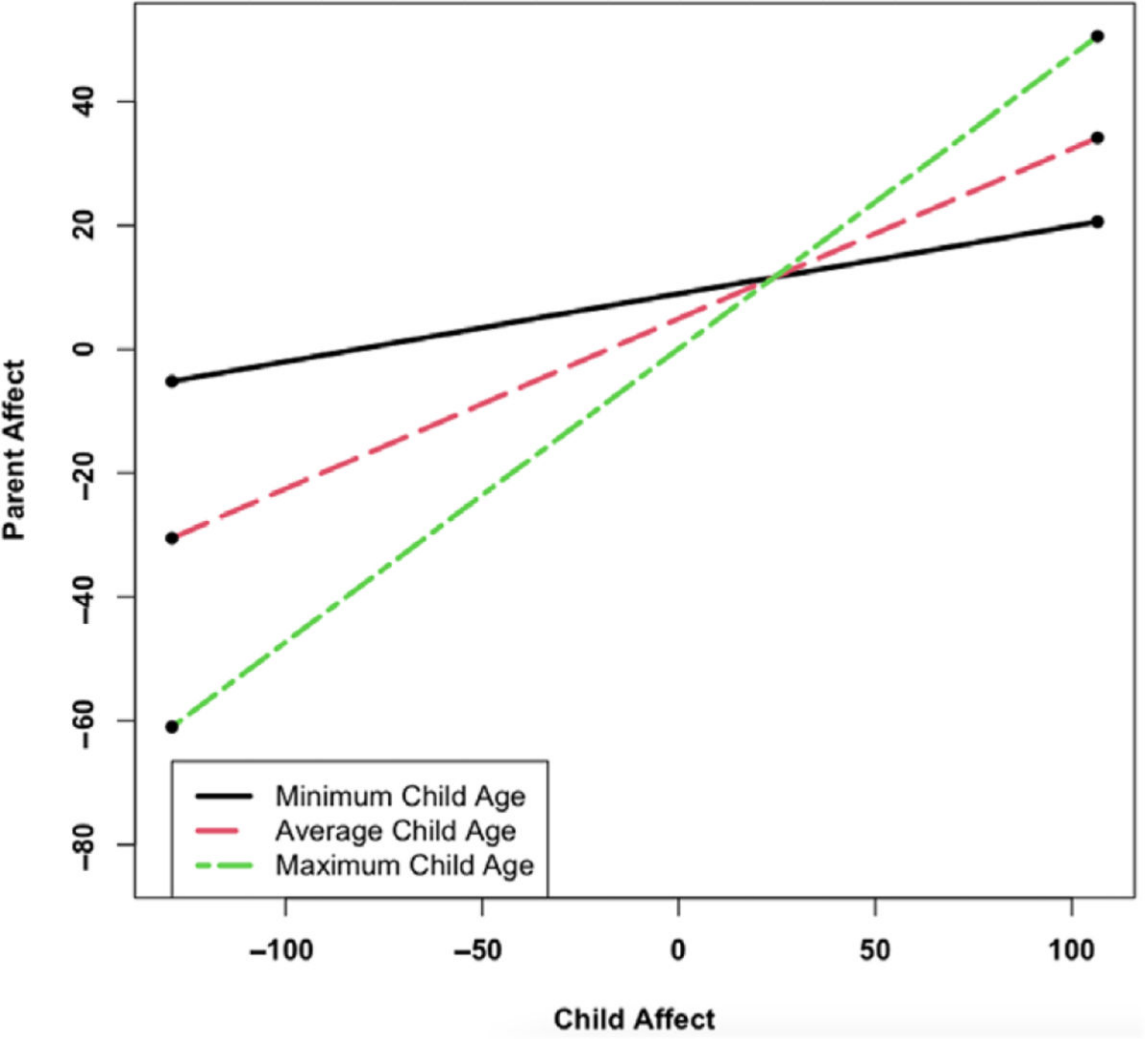
Adolescent Age Moderates the Association between Current Adolescent Affect and Current Parent Affect *Note*. Minimum adolescent age (10 years old): simple slope = 0.11 (0.06), *z* = 1.99, *p* = 0.05. Average adolescent age (12 years old): simple slope = 0.27 (0.04), *z* = 7.67, *p* < .001. Maximum adolescent age (15 years old): simple slope = 0.47 (0.07), *z* = 6.67, *p* < .001. Region of significance: adolescent age > −2.29.

With regard to Aim 2, adolescent age moderated parent–adolescent synchrony, but only in the Parent Affect Model ($\gamma_{11}$ = .07, *p* = .001). As depicted in Figure 1, parent–adolescent synchrony was stronger (i.e., the slope was steeper) when adolescents were older (region of significance: adolescent age > −2.29). Results are displayed in Table 5. Exploratory analyses did not provide evidence of an Adolescent Age × Mean Affect interaction on parent or adolescent affect.

**Figure 2:**
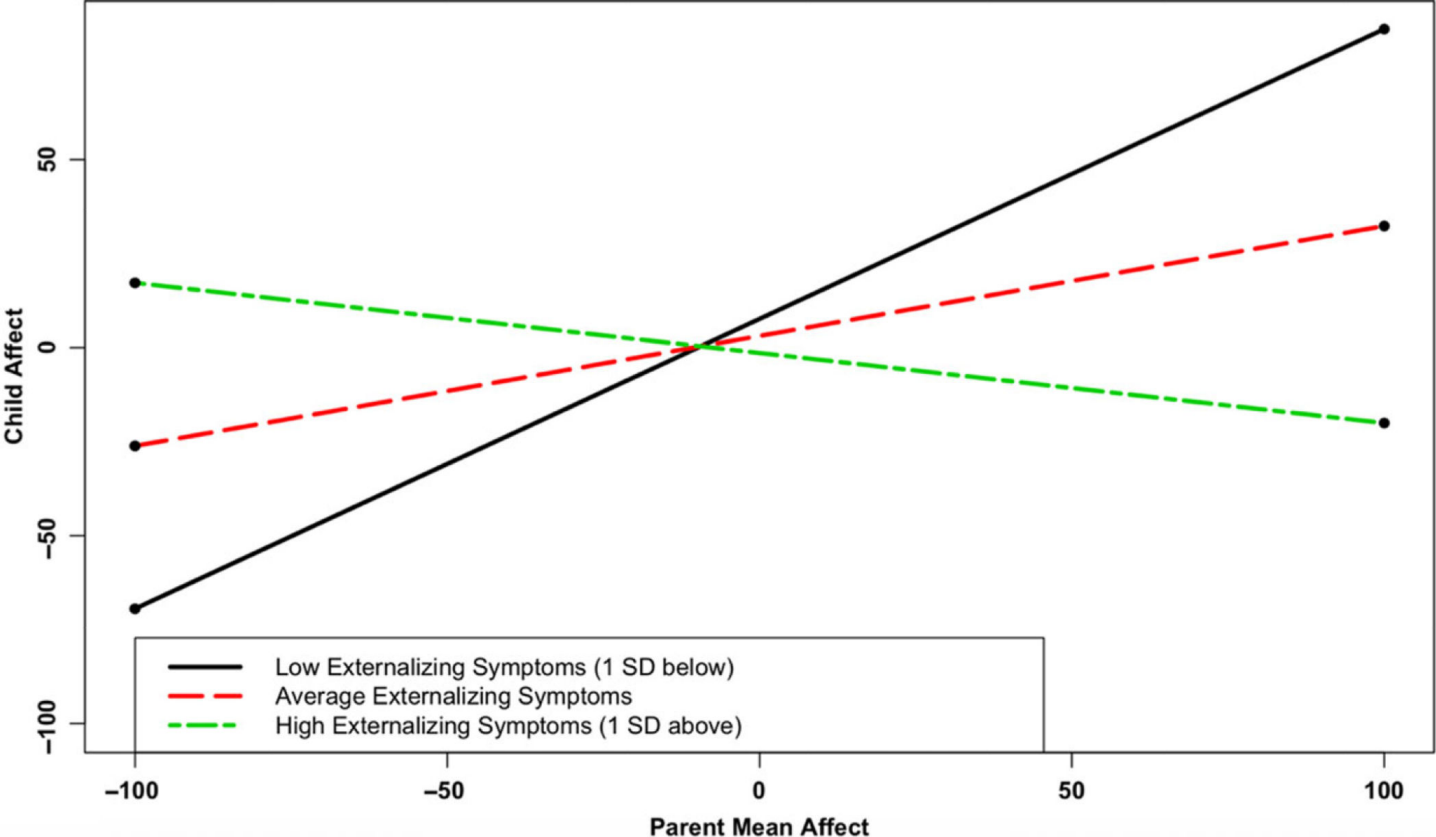
Adolescent Externalizing Symptoms Moderates the Association Between Parent Mean Affect and Current Adolescent Affect *Note*. Low externalizing symptoms (1 SD below average): simple slope = 0.77 (0.21), *z* = 3.68, *p* < 0.001. Average internalizing symptoms: simple slope = 0.29 (0.16), *z* = 1.84, *p* = .07. High internalizing symptoms (1 SD above average): simple slope = −0.19 (0.30), *z* = −0.62, *p* = 0.54. Region of significance: externalizing symptoms < −.22.

**Figure 3:**
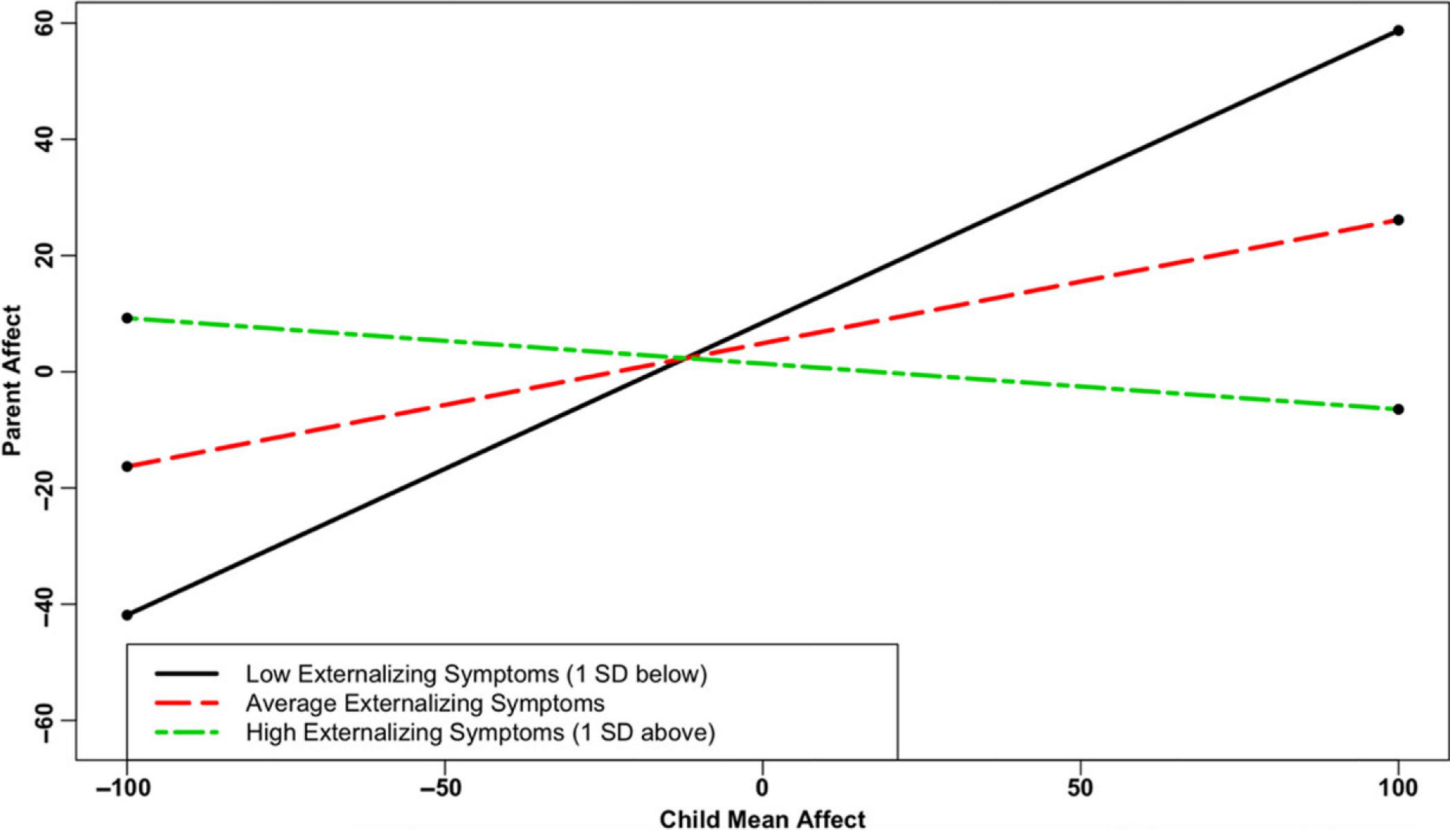
Adolescent Externalizing Symptoms Moderates the Association Between Adolescent Mean Affect and Current Parent Affect *Note*. Low externalizing symptoms (1 SD below average): simple slope = 0.50 (0.14), *z* = 3.54, p < .001. Average internalizing symptoms: simple slope = 0.21 (0.12), *z* = 1.81, *p* = .07. High internalizing symptoms (1 SD above average): simple slope = −0.08 (0.22), *z* = −0.35, *p* = 0.73. Region of significance: externalizing symptoms < −.32.

In Aim 3, we examined how associations between parent and adolescent affect, both in terms of mean affect and affective synchrony, may differ based on levels of adolescent internalizing and externalizing symptoms (Table 5). In partial support of hypothesis 3 (level-2 analyses), externalizing symptoms interacted with mean partner affect to predict affect in both the Adolescent Affect ($\gamma_{07}$ = −.07, *p* = .02) and Parent Affect ($\gamma_{07}$ = −.04, *p* = .05) models. As depicted in Figure 2, parent mean affect predicted adolescent affect when externalizing symptoms were below the mean in the Adolescent Affect Model (region of significance: adolescent externalizing symptoms < −0.22). Similarly, Figure 3 shows that adolescent mean affect predicted parent affect when externalizing symptoms were below the mean in the Parent Affect Model (region of significance: adolescent externalizing symptoms < −0.32).

**Figure 4:**
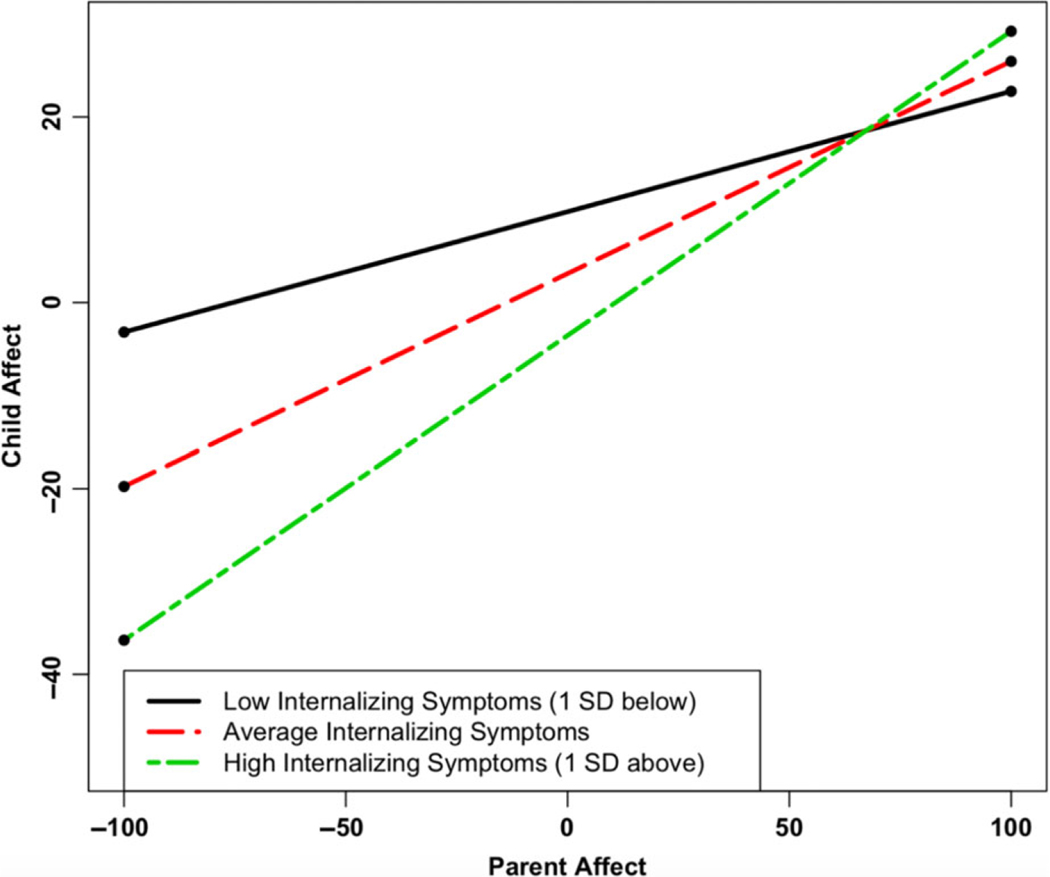
Adolescent Internalizing Symptoms Moderates the Association Between Current Parent Affect and Current Adolescent Affect *Note*. Low internalizing symptoms (1 SD below average): simple slope = 0.13 (0.05), *z* = 2.40, *p* = 0.02. Average internalizing symptoms: simple slope = 0.23 (0.04), *z* = 6.50, *p* < .001. High internalizing symptoms (1 SD below average): simple slope = 0.33 (0.05), *z* = 6.67, *p* < .001. Region of significance: internalizing symptoms > −10.33.

**Figure 5:**
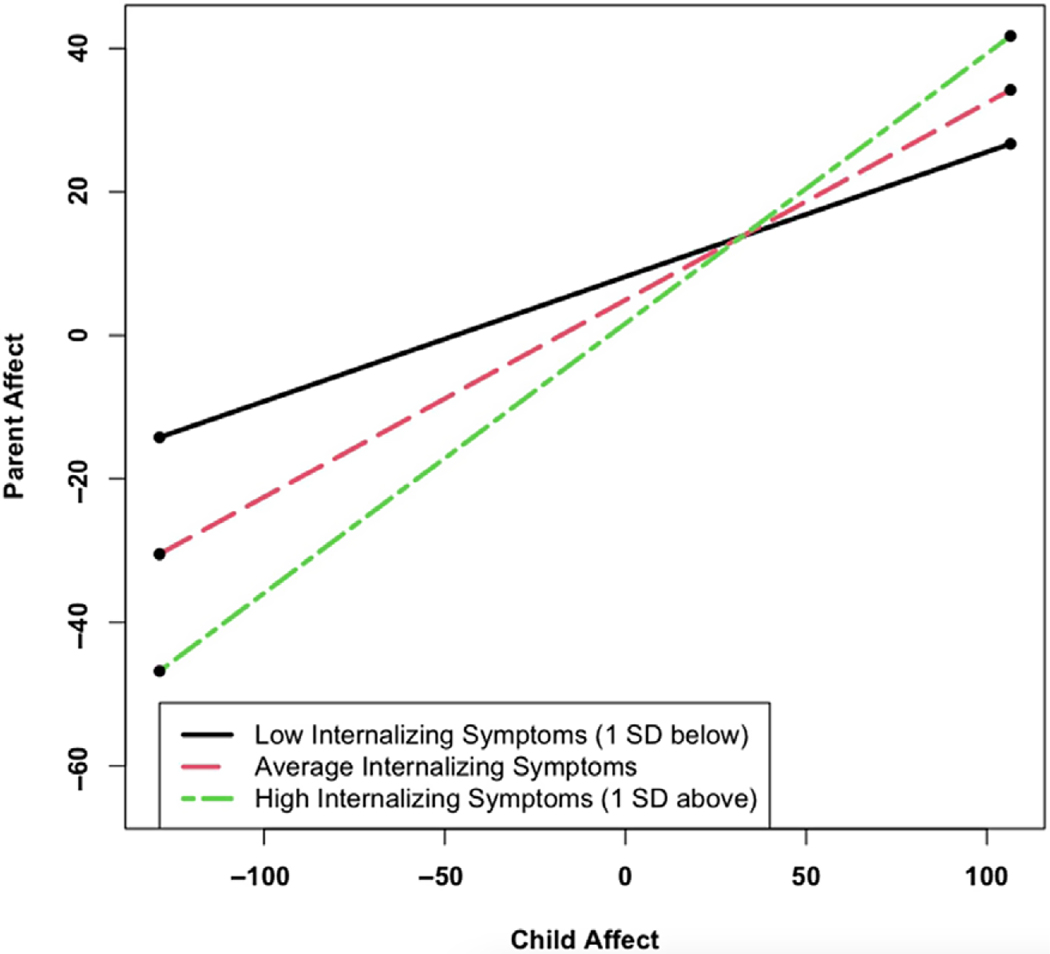
Internalizing Symptoms Moderates the Association Between Current Adolescent Affect and Current Parent Affect *Note*. Low internalizing symptoms (1 SD below average): simple slope = 0.17 (0.05), *z* = 3.24, *p* = .001. Average internalizing symptoms: simple slope = 0.27 (0.04), *z* = 7.67, *p* < .001. High internalizing symptoms (1 SD above average): simple slope = 0.38 (0.05), *z* = 7.59, *p* < .001. Region of significance: internalizing symptoms > −12.84.

Analyses examining the interaction of adolescent internalizing and externalizing symptoms and moment-to-moment affect (level-1 analyses) were exploratory. We found that internalizing symptoms, but not externalizing symptoms, moderated parent–adolescent synchrony. These results were consistent in both the Adolescent Affect Model (γ_12_ = .01, *p* = .01) and Parent Affect Model (γ_12_ = .01, *p* = .01). As depicted in Figures 4 and 5, parent–adolescent synchrony was higher (i.e., the slope was steeper) when the adolescent had more internalizing problems (region of significance: Adolescent Affect Model, internalizing symptoms > −10.33; Parent Affect Model, internalizing symptoms > −12.84).

## Discussion

The parent–child dyadic relationship is an important context in which youth learn about the experience, expression, and regulation of emotions. Dyadic exchanges of emotion begin early in childhood and continue during adolescence. Specifically, parent and child overall affect and moment-to-moment affect are associated in early childhood (Bornstein et al., 2021; Feldman, 2007; Sallquist et al., 2010). However, relatively little research has examined these relations in adolescence (Lougheed, 2020). Understanding affective processes in adolescence may be important, as risk for developing both internalizing and externalizing symptoms increases during this developmental period (Kessler et al., 2007).

The current study used multilevel modeling to examine both level-2 (mean-level affect) and level-1 (affective synchrony) relations in the context of a VMR task following a 10-min conflict-topic discussion. Psychological scientists have predominantly concerned themselves with elucidating sources of variation between individuals. Yet, only under stringent conditions do level-2 effects generalize to level-1 phenomena (Molenaar, 2004). In the current study, mean-level analyses spoke to the affective *content* of parent–child interactions and affective synchrony analyses spoke to affective *structure.* Both level-2 and level-1 effects provided important and unique information. Level-1, but not level-2, associations varied by child age, such that parent–adolescent synchrony was stronger when adolescents were older. Further, we found differential interactions with adolescent psychological symptoms based on level-1 and level-2 affect phenomena; level-1 affect interacted with internalizing symptoms and level-2 affect interacted with externalizing symptoms.

In the current study, we collected self-reported affect ratings using a VMR procedure to minimize potential biases in retrospective recall during a laboratory conflict-topic discussion task that provided similar contexts for emotion generation across dyads. Feasibility of the use of this task with parents and adolescents was supported, considering that no families actively refused to participate in the interaction task or to provide ratings of their emotions using the VMR procedure. Data were obtained from 93% of adolescents and 95% of parents, with missing data largely due to research assistant error. Preliminary construct validity of this task was supported, as well, considering medium positive associations between parent and adolescent affect and small negative associations between adolescent in-task affect and their own internalizing and externalizing symptoms. VMR has been implemented in studies of adult couples (Rohrbaugh et al., 2009), but this is one of the first studies to provide evidence that parents, and most importantly adolescents, can provide affect ratings on a moment-to-moment basis while viewing a video of themselves (see also Main et al., 2021).

### Aim 1: Associations between parent and adolescent affect

As our first aim, we sought to better understand the association between parent affect and adolescent affect over the course of the conflict-topic discussion task. We examined each partner’s mean affect across the interaction (level-2 effects), as well as moment-to-moment changes in each partner’s affect, or affective synchrony (level-1 effects). As hypothesized, mean parent and adolescent affect were positively related to the affect of the other member of the dyad. Further, as expected, we found positive associations between partners’ concurrent, moment-to-moment affect. In other words, we found both level-2 and level-1 effects in affective interactions between parents and adolescents. These findings indicate that the correspondence in overall affect and affective synchrony that has been documented during interactions between parents and infants and young children (e.g., Bornstein et al., 2021; Feldman, 2007; Sallquist et al., 2010) is also present in early to mid-adolescence. Previous reviews have highlighted the importance of examining affective synchrony in parent–adolescent exchanges (e.g., Lougheed, 2020) and the current study is one of the first examples to do so (see also Main et al., 2021). Further, the current findings suggest that VMR is a potentially important tool to capture affect in parent–adolescent dyads in real time.

### Aim 2: Parent–adolescent affective processes as a function of youth age

The second aim of the study was to examine how parent–child affective patterns may be related to adolescent age. Our sample consisted of 10- to 15-year-olds, and so we were able to explore associations in early to mid-adolescence, specifically. We found significant associations between adolescent age and parent–adolescent affective synchrony, with the level of affective synchrony strongest for the oldest adolescents in our sample. The pattern of increasing synchrony with older age, which corresponds to mid-adolescence in the current sample, reflects the transitional nature of adolescence. Patterns of dyadic exchange that become well established throughout childhood are disrupted in early adolescence and restabilize over time (Granic et al., 2003). To negotiate changes in early adolescence, parents may scaffold regulation through greater maintenance of neutral states while youth experience extreme positive or negative affect (reflected through less synchrony; van Bommel et al., 2019). Parents of older adolescents may engage in less scaffolding and greater matching of affective states, and reciprocally, youth may have developed skills to regulate their emotional experience to match their parents’ affect (Branje, 2018). Future research may benefit from recruiting samples with wider age ranges (e.g., childhood through late adolescence) to better understand how parent–child affective synchrony waxes and wanes across development. Of note, effects emerged in the Parent Affect Model but not the Adolescent Affect Model, and they should be tested and replicated in future research.

We did not find significant associations between adolescent age and mean levels of either adolescent or parent affect. In their meta-analysis, Laursen et al. (1998) found a small increase in intensity of conflict affect between early and mid-adolescence, but only for father–son dyads. Relatedly, one potential explanation for the observed findings is that overall affect during a conflict discussion is more negative for dyads with adolescents at the upper range of our age group than those at the middle and lower ranges, but only for certain gendered pairs (e.g., father–son dyads). The parents in our sample are primarily (93%) mothers, precluding separate analyses for mothers and fathers. Future research will benefit from samples that are larger, more heterogeneous, and more balanced allowing for the examination of parent–adolescent affect dynamics as a function of both age and gender.

### Aim 3: Parent–adolescent affective processes as a function of youth internalizing and externalizing symptoms

Finally, we were interested in the ways in which associations between parent and adolescent affect may differ based on levels of adolescent internalizing and externalizing symptoms. We hypothesized that mean affect for both parents and adolescents would be more negative when levels of adolescent internalizing and externalizing symptoms were higher. We had no a priori hypothesis for the association between parent–child affective synchrony and adolescent internalizing and externalizing symptoms. An interesting pattern of findings emerged.

More positive adolescent mean affect was associated with more positive parent affect (and vice versa), but only for dyads in which adolescents reported low externalizing problems. For dyads in which adolescents reported average or high externalizing problems, there was no association between adolescent and parent mean affect (and vice versa). In adolescence, parents might react in several ways to youth with externalizing symptoms during a conflict discussion, such as through escalation of negative affect, permissiveness, or inconsistency (Granic & Patterson, 2006). Variation in parental responsiveness in this subgroup of youth may contribute to nonsignificant associations between parent and child mean affect. Although likely due to reduced power to detect a very small effect, the observed association was nonsignificant ($\gamma_{07}$ = –.04 *p* = .09) in a subsample of only mothers (i.e., excluding fathers; *n* = 51). As such, this finding should be interpreted with caution and replicated in future work.

We did not find support for the interaction of externalizing symptoms and affective synchrony. Lunkenheimer et al. (2011) found associations between parent–child affective flexibility and youth externalizing symptoms to depend on parent gender, such that affective flexibility was associated with more symptoms in mother–child dyads and fewer symptoms in father–child dyads. It is possible that a similar effect could emerge for affective synchrony, again underscoring the importance of careful sample selection in future studies that will allow researchers to probe these processes.

Levels of parent–adolescent affective synchrony were higher for dyads in which adolescents reported higher levels of internalizing problems. The presence of internalizing symptoms in youth may prompt both youth and parents to display greater emotional acceptance (Creavy et al., 2020) or empathy for the other (Tone & Tully, 2014), resulting in more time in joint negative or positive affective states. Children of depressed and anxious parents are at increased risk for developing internalizing symptoms in adolescence (Goodman, 2020; Eley et al., 2015), and so parental psychopathology in dyads characterized by high adolescent internalizing symptoms may further contribute to shared time in negative affective states (Henry et al., 2020). Associations among parental psychopathology, adolescent psychopathology, and affect dynamics in parent–child interactions were not tested in the current study, however, and this remains an area for future research.

We did not find support for our hypothesis that parent and adolescent mean affect would be more negative when levels of adolescent internalizing symptoms were higher. A study by Schwartz et al. (2014) may provide context for these findings. Schwartz et al. (2014) found the onset of adolescent depression to be predicted by higher rates of maternal aggressive behavior in an event planning task (i.e., designed to elicit positive emotion) and lower rates of positive behavior during a problem-solving task (i.e., designed to elicit negative emotion). In discussing these findings, the authors highlighted the importance of context (here, the type of interaction task) in determining the predictive value of observed behavior and emotion in interaction tasks. Following this line of reasoning, in the current study, higher levels of negative emotion in the conflict discussion interaction task may be functional, normative, and unrelated to youth internalizing symptoms during conflict discussions. Our nonsignificant findings are also consistent with typical increases in negative affect during parent–child conflict discussions during adolescence (Granic et al., 2003; Laursen et al., 1998). Still, testing the specificity of the associations of emotions with internalizing versus externalizing symptoms is a priority for future research.

### Limitations of the current study and areas for future research

These novel findings notwithstanding, the current study also had limitations which provide directions for future research. First, concurrent covariation may reflect the transfer or exchange of emotions between partners, but concurrent covariation may also be influenced by third variables such as shared experiences (Butler, 2011). For example, being in the laboratory together may have influenced synchrony and mean affect in both parents and adolescents (Bronfenbrenner, 1979). Examining emotion dynamics in daily life is an important next step for synchrony research, as estimating the convergence of multiple methods for measuring dyadic affect dynamics (i.e., experience sampling paradigms with laboratory methods and questionnaires) will further clarify dyadic affect dynamics as a construct (Cole et al., 2020). Experience sampling paradigms provide numerous possibilities for self-report, observational coding, and amalgams of the two methods. Researchers might ask parents and their adolescents to rate their own affect multiple times over the course of the day, or a single interaction, using an abbreviated version of the Positive and Negative Affect Schedule (Watson et al., 1988) delivered through smartphones (e.g., Rogers et al., 2018). Passive audio recordings (e.g., using an Electronically Activated Recorder or smartphone technology) might be collected from parent and child and subsequently rated by objective coders on a moment-to-moment basis (e.g., Imami et al., 2015). Similar to the current study, an observational self-report method might be used, such that parents and adolescents use VMR methods to rate audio recordings collected in daily life upon returning to the laboratory. Further, researchers might implement multiple methods (self-report, observations, observational self-report) in a single experience sampling study.

Second, our rating scale incorporates affect valence and intensity together on a continuum ranging from very negative (−100) to very positive (100), and we were unable to test our hypotheses separately for positive and negative affect. As described by Hollenstein et al. (2017) and exemplified in the current study, the structural patterns of affect alone demonstrate associations with youth functioning in various stages of development. Still, future research might implement study designs and data analytic techniques that allow for the differentiation of affect valence (i.e., positive vs. negative) and emotion type (e.g., sad vs. angry) to develop a more nuanced understanding of mean-level affect and affective synchrony in parent–child relationships (e.g., Main et al., 2016).

Third, we examined concurrent synchrony, which presupposes that parent and adolescent emotion channels are tightly coupled. In contrast, it could be that emotion channels are lagged, such that current emotions are most strongly influenced by the partner’s prior emotions (perhaps 3, 5, or 10 s prior). Further, synchrony (concurrent and lagged) is only one of several ways to understand dynamic interpersonal affective systems. The field would benefit from future research on possible lagged effects of partners’ emotions, as well as other structural aspects of affective exchange in parent–adolescent conflict discussions (e.g., flexibility, phase transitions) (see Butler, 2011).

Fourth, the magnitude of the multilevel model variance at levels 1 and 2 indicates that additional predictors can be added. Future research should examine parent and dyadic factors, in addition to adolescent factors, that may influence mean affect and affective synchrony in parent–adolescent dyads (van Bommel et al., 2019). For example, our study was primarily comprised of mothers (93%) and results may not generalize to father–child interactions. As mentioned earlier in the Discussion, samples that are larger, more heterogeneous, and more balanced will allow for the exploration of individual differences in parent–child affect dynamics. Further, study designs would benefit from the integration of self-report with physiological methods to better understand affective synchrony as a whole-body process (DePasquale, 2020; Krzeczkowski et al., 2020; Quiñones-Camacho et al., 2020). Nevertheless, results provide initial evidence for synchrony in moment-to-moment parent–adolescent affect in adolescence and highlight the potential for the use of VMR as a method to capture affective experience in adolescents and their parents.

Finally, while otherwise informative, the current cross-sectional investigation cannot point to whether high versus low mean-level positive/negative affect, or synchrony versus asynchrony, in parent–adolescent interactions is developmentally adaptive or maladaptive. Future work incorporating longitudinal measurement burst designs will elucidate how affective components of parent–child interactions change and remain stable across development and predict long-term adjustment (Lougheed & Keskin, 2021). Nevertheless, as is true with many psychological phenomena, the picture is likely far more nuanced than “synchrony is good, and asynchrony is bad” or the reverse. As we have alluded to throughout the Discussion, understanding additional moderators of effects will be important, including parent and child gender, parent psychopathology, and task demands. Incorporating measures of individual differences in core affective skills upon which dyadic interactions rely, namely emotion recognition and regulation (including flexibility, see Lougheed & Hollenstein, 2016), will be essential. VMR might facilitate these efforts. For example, participants could continuously rate their partner’s affective state after rating their own affective state, and they could provide narrative reports of what they were doing or thinking to deal with their emotions while reviewing clips of their interaction to be later coded by emotion regulation/coping factor or strategy by independent raters.

### Implications and conclusions

The current study supported the feasibility of using VMR with parents and adolescents to retroactively rate affect generated in conflict-topic discussions. Similar methods have been used to bring dyadic behavioral and affective dynamics into the awareness of caregivers of infants and young children in the context of interventions. Attachment and Biobehavioral Catch-up (Dozier et al., 2006) and Filming Interactions to Nurture Development (Fisher et al., 2016) incorporate the review of videorecords to help shape sensitive caregiving behaviors and prevent later behavioral and emotional problems in youth (Balldin et al., 2018; Grube & Liming, 2018; Fukkink, 2008). Extending these programs into adolescence may be warranted; that is, parents *and* adolescents could be coached to observe and understand moment-to-moment changes in the emotion channels of themselves and their partner. Observation of dyadic interactions will provide an important context from which dyads can work together to build skills in emotion recognition and regulation.

Parents and children are linked across time, as are their emotions. Affective exchanges that occur over countless short-term dyadic interactions are key to shaping long-term emotional processes and psychological functioning. The results of the current study are a window into the content and structure of affect in parent–child interactions during early to mid-adolescence.

## Supplementary Material

1

## Figures and Tables

**Table 1.** Sociodemographic characteristics of adolescents and parents

	Characteristic	*M/%*	*SD*
Adolescent	Age	12.27	1.67
	Gender (% female)	54.55	
	Race/ethnicity (%)		
	White	69.09	
	Black	23.64	
	Asian	5.45	
	More than one race/other	1.82	
Parent	Age	40.00	5.45
	Gender (% female)	92.72	
	Education (%)		
	Graduate degree	37.04	
	Some graduate education	9.26	
	College graduate	29.63	
	Some college or technical school	22.22	
	High school graduate	1.85	
	Marital status (%)		
	Married/living with partner	69.09	
	Never married	14.55	
	Divorced or annulled	12.73	
	Separated	1.82	
	Widowed	1.82	

**Table 2.** Means, standard deviations, ranges, and bivariate correlations for study variables

Variable	*M*	*SD*	Range	1	2	3	4
1. Adolescent affect	4.23	44.13	[−100, 100]	−			
2. Parent affect	5.65	41.78	[−100, 100]	0.31***	—		
3. Adolescent internalizing symptoms	15.15	9.06	[2, 41]	−0.26***	−0.18***	—	
4. Adolescent externalizing symptoms	11.29	7.40	[0, 33]	−0.18***	−0.19***	0.39***	—

*Note*. Raw scores are presented for adolescent internalizing symptoms and adolescent externalizing symptoms.

*** *p*<.001.

**Table 3.** Null models predicting adolescent affect and parent affect

Dependent variable	Independent variable	PE	*SE*	*P*
Adolescent affect	Fixed effects			
	Intercept ($\gamma_{00}$)	5.18	4.39	.25
	Random effects			
	Intercept ($\tau_{00}$)	944.83	192.41	
	Residual $\left(\sigma_{e}^{2}\right)$	1032.45	26.88	
	ICC	48%		
Parent affect	Fixed effects			
	Intercept ($\gamma_{00}$)	5.65	3.59	.12
	Random effects			
	Intercept ($\tau_{00}$)	666.98	133.06	
	Residual $\left(\sigma_{e}^{2}\right)$	1077.81	27.26	
	ICC	38%		

*Note*. PE = parameter estimate; SE = standard error; ICC = intraclass correlation coefficient.

**Table 4.** Multilevel models predicting adolescent affect and parent affect

Dependent variable	Independent variable	PE	*SE*	*p*
Adolescent affect	Fixed effects			
	Intercept ($\gamma_{00}$)	1.33	4.01	.74
	Parent affect_t_^a^ ($\gamma_{10}$)	0.24	0.04	<.001
	Parent affect mean ($\gamma_{01}$)	0.56	0.15	.001
	Random effects			
	Intercept ($\tau_{00}$)	733.57	149.95	
	Parent affect_t_^a^ ($\tau_{11}$)	0.04	0.01	
	Residual $\left(\sigma_{e}^{2}\right)$	960.37	25.22	
Parent affect	Fixed effects			
	Intercept ($\gamma_{00}$)	5.08	3.22	.12
	Adolescent affect_t_^a^ ($\tau_{10}$)	0.28	0.04	<.001
	Adolescent affect mean ($\gamma_{01}$)	0.35	0.10	.001
	Random effects			
	Intercept ($\tau_{00}$)	498.98	101.44	
	Adolescent affect_t_^a^ ($\tau_{11}$)	0.06	0.02	
	Residual $\left(\sigma_{e}^{2}\right)$	974.78	25.63	

*Note*. PE = parameter estimate; SE = standard error; Adolescent affect_*t*_ = adolescent affect at the current interval; Parent affect_*t*_ = parent affect at the current interval.

a Person mean centered.

**Table 5.** Multilevel models predicting adolescent affect and parent affect, including interactions

Dependent variable	Independent variable	PE	SE	*P*
Adolescent affect	Fixed effects			
	Intercept ($\gamma_{00}$)	3.13	3.74	.41
	Parent affect_t_^a^ ($\gamma_{10}$)	0.23	0.04	<.001
	Parent affect mean ($\gamma_{01}$)	0.29	0.16	.07
	Adolescent Age ($\gamma_{02}$)	−3.32	2.10	.12
	Adolescent Age × Parent affect_t_^a^ ($\gamma_{11}$)	−0.01	0.02	.48
	Adolescent Age × Parent affect mean ($\gamma_{05}$)	0.004	0.10	.97
	Internalizing ($\gamma_{03}$)	−0.74	0.42	.08
	Internalizing × Parent affect_t_^a^ ($\gamma_{12}$)	0.01	0.004	.01
	Internalizing × Parent affect mean ($\gamma_{06}$)	0.02	0.02	.32
	Externalizing ($\gamma_{04}$)	−0.63	0.61	.31
	Externalizing × Parent affect_t_^a^ ($\gamma_{13}$)	−0.007	0.005	.17
	Externalizing × Parent affect mean ($\gamma_{07}$)	−0.07	0.03	.02
	Random effects			
	Intercept ($\tau_{00}$)	575.49	118.36	
	Parent affect_t_^a^ ($\tau_{11}$)	0.03	0.01	
	Residual $\left(\sigma_{e}^{2}\right)$	959.87	25.20	
Parent affect	Fixed effects			
	Intercept ($\gamma_{00}$)	4.92	3.32	.15
	Adolescent affect_t_^a^ ($\gamma_{10}$)	0.27	0.04	<.001
	Adolescent affect mean ($\gamma_{01}$)	0.21	0.12	.08
	Adolescent Age ($\gamma_{02}$)	−1.77	1.87	.35
	Adolescent Age × Adolescent affect_t_^a^ ($\gamma_{11}$)	0.07	0.02	.001
	Adolescent Age × Adolescent affect mean ($\gamma_{05}$)	0.01	0.06	.83
	Internalizing ($\gamma_{03}$)	−0.36	0.39	.36
	Internalizing × Adolescent affect_t_^a^ ($\gamma_{12}$)	0.01	0.004	.01
	Internalizing × Adolescent affect mean ($\gamma_{06}$)	0.002	0.02	.88
	Externalizing ($\gamma_{04}$)	−0.49	0.49	.32
	Externalizing × Adolescent affect_t_^a^ ($\gamma_{13}$)	−0.008	0.006	.14
	Externalizing × Adolescent affect mean ($\gamma_{07}$)	−0.04	0.02	.05
	Random effects			
	Intercept ($\tau_{00}$)	439.72	91.49	
	Adolescent affect_t_^a^ ($\tau_{11}$)	0.03	0.01	
	Residual $\left(\sigma_{e}^{2}\right)$	975.89	25.69	

*Note*. PE = parameter estimate; SE = standard error; Parent affect_*t*_ = parent affect at the current interval; Adolescent affect_*t*_ = adolescent affect at the current interval.

a Person mean centered.

## Footnotes

1 As described above, affective synchrony has been quantified in several ways (Butler, 2011). In the present study, synchrony reflects the concurrent covariation of adolescent and parent affect using a multilevel modeling approach.

2 Analyses testing the study hypotheses were conducted separately for the full sample (*N* = 55) and a subsample excluding fathers (i.e., mothers only, *n* = 51). The majority of the findings were the same in both samples, and so we present results from the full sample here. However, the Externalizing X Adolescent Affect Mean effect in the full sample (*γ*_07_ = −.04 *p* = .05) became nonsignificant in the subsample (*γ*_07_ = −.04 *p* = .09), likely due to reduced power to detect this very small effect. Potential implications are presented in the Discussion.

3. The basic Adolescent Affect and Parent Affect Models are roughly (imprecision will occur due to centering and differential missing data) statistically equal. Nevertheless, we analyzed both equations and present their results (Table 4) as they provide the basis for Aims 2 and 3.

4 Youth outside of the age range (<11 years old) for the YSR (*n* = 12) had similar *T*-scores for internalizing symptoms (*M* = 55.00, *SD* = 10.72) and externalizing symptoms (*M* = 49.58, *SD* = 6.96). Group differences were nonsignificant for internalizing symptoms, *t*(53) = −0.55, *p* = .59 and externalizing symptoms, *t*(53) = −1.13, *p* = .26.
