# Supplementary material for: Mean-level correspondence and moment-to-moment synchrony in adolescent and parent affect: Exploring associations with adolescent age and internalizing and externalizing symptoms
Source: Dev Psychopathol. Author manuscript; Available in PMC 2023 Nov 1. (PMC9881583; doi:10.1017/S0954579422000062)
Supplement: 1 [file NIHMS1772385-supplement-1.docx]

**Supplementary Information**

***Null Models (Table 3)***

**Adolescent Affect Model**

Level 1 equation: ${AA}_{td}= \beta_{0d}+ e_{td}$

Level 2 equation: $\beta_{0d}= \gamma_{00}+ u_{0d}$

Mixed model: ${AA}_{td}= \gamma_{00}+ u_{0d}+ e_{td}$

$e_{td}\sim N(0,\sigma_{e}^{2}$)

$$u_{0d}\sim N(0, \tau_{00})$$

**Parent Affect Model**

Level 1 equation: ${PA}_{td}= \beta_{0d}+ e_{td}$

Level 2 equation: $\beta_{0d}= \gamma_{00}+ u_{0d}$

Mixed model: ${PA}_{td}= \gamma_{00}+ u_{0d}+ e_{td}$

$e_{td}\sim N(0,\sigma_{e}^{2}$)

$$u_{0d}\sim N(0, \tau_{00})$$

***Multilevel Model Predicting Parent Affect (Table 4)***

Level 1 equation: ${PA}_{td}= \beta_{0d}+ \beta_{1}{(AA}_{td}-\bar{AA}_{.d}){+ e}_{td}$

Level 2 equation: $\beta_{0d}= \gamma_{00}+\gamma_{01}\bar{AA}_{.d}+u_{0d}$

$\beta_{1d}$ = $\gamma_{10}$ $+ u_{1d}$

Mixed model: ${PA}_{td}= \gamma_{00}$ + $\gamma_{01}\bar{AA}_{.d}$ + $\gamma_{10}{(AA}_{td}-\bar{AA}_{.d})$ +$u_{0d}$ $+ u_{1d}{(AA}_{td}-\bar{AA}_{.d})$

$e_{td}\sim N(0,\sigma_{e}^{2}$)

$$\left[ \begin{aligned} u_{0d} \\ u_{1d} \end{aligned} \right]\sim N \left( \left[ \begin{aligned} 0 \\ 0 \end{aligned} \right],\left[ \begin{matrix} \tau_{00} & \\ \tau_{10} & \tau_{11} \end{matrix} \right] \right)$$

***Multilevel Model Predicting Parent Affect, Including Interactions (Table 5)***

Level 1 equation: ${PA}_{td}= \beta_{0d}+ \beta_{1}{(AA}_{td}-\bar{AA}_{.d}){+ e}_{td}$

Level 2 equation: $\beta_{0d}= \gamma_{00}+\gamma_{01}\bar{AA}_{.d}+\gamma_{02}{age}_{d}+\gamma_{03}{intern}_{d}+\gamma_{04}{extern}_{d}+\gamma_{05}{age}_{d}\bar{AA}_{.d}+\gamma_{06}{intern}_{d}\bar{AA}_{.d}+\gamma_{07}{extern}_{d}\bar{AA}_{.d}+ u_{0d}$

$\beta_{1d}$ = $\gamma_{10}+\gamma_{11}{age}_{d}+\gamma_{12}{intern}_{d}+\gamma_{13}{extern}_{d}+ u_{1d}$

Mixed model: ${PA}_{td}= \gamma_{00}$ + $\gamma_{01}\bar{AA}_{.d}$ $+\gamma_{02}{age}_{d}$+ $\gamma_{03}{intern}_{d}+\gamma_{04}{extern}_{d}+\gamma_{05}{age}_{d}\bar{AA}_{.d}+ \gamma_{06}{intern}_{d}\bar{AA}_{.d}+\gamma_{07}{extern}_{d}\bar{AA}_{.d}+$ $\gamma_{10}{(AA}_{td}-\bar{AA}_{.d})$ + $\gamma_{11}{age}_{d}{(AA}_{td}-\bar{AA}_{.d})+\gamma_{12}{intern}_{d}{(AA}_{td}-\bar{AA}_{.d})$ + $\gamma_{13}{extern}_{d}{(AA}_{td}-\bar{AA}_{.d})+ u_{0d}$ $+ u_{1d}{(AA}_{td}-\bar{AA}_{.d})$

$e_{d}\sim N(0,\sigma_{e}^{2}$)

$$\left[ \begin{aligned} u_{0d} \\ u_{1d} \end{aligned} \right]\sim N \left( \left[ \begin{aligned} 0 \\ 0 \end{aligned} \right],\left[ \begin{matrix} \tau_{00} & \\ \tau_{10} & \tau_{11} \end{matrix} \right] \right)$$
